# Symptom patterns of comorbid depression and anxiety among older adults in China and their predictors

**DOI:** 10.1002/pchj.729

**Published:** 2024-01-24

**Authors:** Lingling Yu, Chuqian Chen

**Affiliations:** ^1^ Department of Philosophy and Science Southeast University at Nanjing Nanjing China; ^2^ Department of Medical Humanities Southeast University at Nanjing Nanjing China

**Keywords:** comorbid depression and anxiety, elderly people, intimate relationship, latent profile analysis

## Abstract

Comorbid depression and anxiety causes serious psychological and physiological damage for older people. This study aimed to identify heterogeneous classes of comorbid depression and anxiety (CDA) among older people in China and to ascertain predictors of latent class membership. Cross‐sectional data of 10,919 cases were extracted from the Chinese Longitudinal Healthy Longevity Survey. Latent profile analysis (LPA) was used to identify symptom patterns of comorbid depression (measured by the 10‐item Center for Epidemiologic Studies Depression Scale) and anxiety (measured by the Generalized Anxiety Disorder 7‐item Scale). Multinomial logistic regressions following bivariate analyses were used to explore the relationship between the derived classes and individual‐ and social‐level factors. Four patterns of CDA were identified: low symptoms of depression and anxiety (30.52%; *n* = 3333), mild depression only (53.26%; *n* = 5815), moderate depression and anxiety (13.82%; *n* = 1509), and severe depression and anxiety (2.40%; *n* = 262). Older people who are male, suffer from multimorbidity, and lack a healthy lifestyle are more likely to have problematic symptom profiles. While intimate relationships with partners and children significantly predicted CDA patterns, the effects of sibling relationships, daily life, and emotional support from the community were insignificant. LPA identified four distinct CDA patterns among a representative sample of older Chinese people. While restless sleep, lack of positive emotions, uselessness, and weak concentration are salient across all profiles, “difficult to relax” is prominent in profiles high in anxiety. In addition to individual‐level variables, social‐level factors, especially intimate relationships with partners and children rather than general links to siblings or the community, have unneglectable impacts on whether and to what extent older Chinese adults suffer from CDA in the cultural context of relationism, patriarchy, and filial piety.

## BACKGROUND

### Comorbid depression and anxiety (CDA) patterns in the ageing population

In a rapidly ageing world (Beekman et al., 2000; Nations, 2022), increasing attention is being paid to the mental health of elderly individuals. Mental health issues that are prevalent among older adults include depression, anxiety, posttraumatic stress, and substance misuse (Cleary et al., 2017; Reynolds et al., 2015; Smith, 2014). Comorbid depression and anxiety (CDA) is a common and significant issue in later life (Byrne & Pachana, 2010). Previous studies unveiled varied prevalence of CDA across different populations. An estimated 26.1%–47.5% of Dutch people (aged 55–85) were found to have CDA, and 28.9% of US people (aged ≥60) who sought anxiety treatment also had depression (Porensky et al., 2009). CDA not only increases the difficulty of treatment compared with either condition alone but also causes more serious psychological and physiological damages, including an increased risk of suicide (Beekman et al., 2000; Tiller, 2013). To achieve a better understanding of late‐life CDA and develop more efficient treatments, an increasing number of scholars around the world are striving to identify heterogeneous subgroups of CDA among elderly people and exploring predictors of subgroup membership.

Latent profile analysis (LPA), a person‐centred method to identify latent subgroups with various outcome patterns (Mori et al., 2020), has been applied to samples from different countries. Among 8504 Irish individuals (aged ≥50) (Curran et al., 2019), three subgroups with various CDA profiles were identified: the comorbidity group (*n* = 1616, 19%), the asymptomatic group (*n* = 3742, 44%), and the group with mild depressive symptoms (*n* = 3146, 37%). Further analysis found that age, sex, education, religious attendance, social network, and time spent abroad are all significant predictors of CDA profiles. In addition, Curran et al. (2022) applied LPA separately to males and females among 7040 English people (aged ≥50) during the COVID‐19 pandemic, and three classes without statistically significant sex differences were uncovered: comorbid depression and anxiety (female: 19.9%; male: 12.8%), depression and subthreshold anxiety (female: 31.6%; male: 29.6%), and no or low depression and anxiety (female: 48.5%; male: 57.6%). Multinomial logistic regression revealed that CDA patterns are associated not only with worrying about finances, the availability of essentials, and health and social care services but also with loneliness and prepandemic mental health status. To the best of our knowledge, latent CDA patterns have never been identified among older populations in China.

### CDA among Chinese elderly people

China is facing severe challenges related to population ageing (Statistics, 2021). It is estimated that by 2050, people aged ≥60 will account for 35.6% of the total population (United Nations, 2022). The prevalence of CDA is approximately 8.7% among people aged ≥60, while the subgroup patterns remain unknown (Zhao et al., 2020). As culture plays a significant role in shaping the manifestation of comorbid patterns (Chentsova‐Dutton & Tsai, 2009), findings from other parts of the world should not be directly applied to the Chinese population. To enable in‐depth theoretical explorations, inform better policy, and generate tailored interventions, it is essential to explore latent CDA patterns and identify predictors of such patterns among elderly individuals in China.

Despite the fact that China is a country with a high degree of heterogeneity owing to its large population and vast geographical scope, previous explorations of CDA among Chinese elderly people were limited to certain areas. For instance, a study on the relationship between life expectancy and CDA (Wu et al., 2021) was based in Jiangxi Province, and an analysis of how CDA is linked to frailty was conducted in West China (W. Y. Zhao et al., 2020). To generate findings regarding CDA patterns and influencing factors that better represent the whole population, it is imperative to analyse data from nationally representative samples.

Unlike the individualist culture that dominates in many parts of the world, the Chinese culture, which is characterized by relationism, gives more weight to relationships (Huang et al., 2018; Wang & Liu, 2010). For instance, a study found that elders (aged ≥65) in China who had closer child–parent relationships had fewer depressive symptoms, while such a link was nonsignificant among non‐Hispanic White Americans (P. Lu et al., 2023). Under such circumstances, social factors are significant shaping forces for the mental health of Chinese older people, thus making it vital to systematically explore how social factors, in addition to individual ones, influence Chinese elderly individuals' CDA patterns. Previous studies on influencing factors of CDA among elderly individuals mainly focused on individual‐level factors, including physical condition (e.g., impaired hearing, long‐term illness), lifestyle (e.g., smoking, drinking), and religion (Curran et al., 2019, 2020, 2022). Only single variables, such as social network, spousal support or strain, and activities of elderly people, were measured to reflect the social dimension (Curran et al., 2020), and no study has systematically explored a series of predictors that belong to the social level. At the social level, both intimate and social relationships should be given serious attention. In traditional Chinese culture, great significance was attached to filial piety and family relationships, and integrated intimate relationships, especially intergenerational links, served as key protectors for the mental health of elderly people (X. Liu et al., 1995; Silverstein et al., 2006). During the past 10 years, however, community services have been considered a supplement to the traditional family care model (Yue et al., 2021), and their use has been found to be inversely related to psychological problems among empty‐nest elders in China (He et al., 2013).

### The present study

The present study aims to identify subgroups of CDA among elderly Chinese individuals and explore predictors of latent class membership. To fill the gaps in previous studies, nationally representative samples were used, and the exploration of predictive factors was based on a systematic framework that consists of both individual‐level predictors and social‐level factors.

**FIGURE 1:**
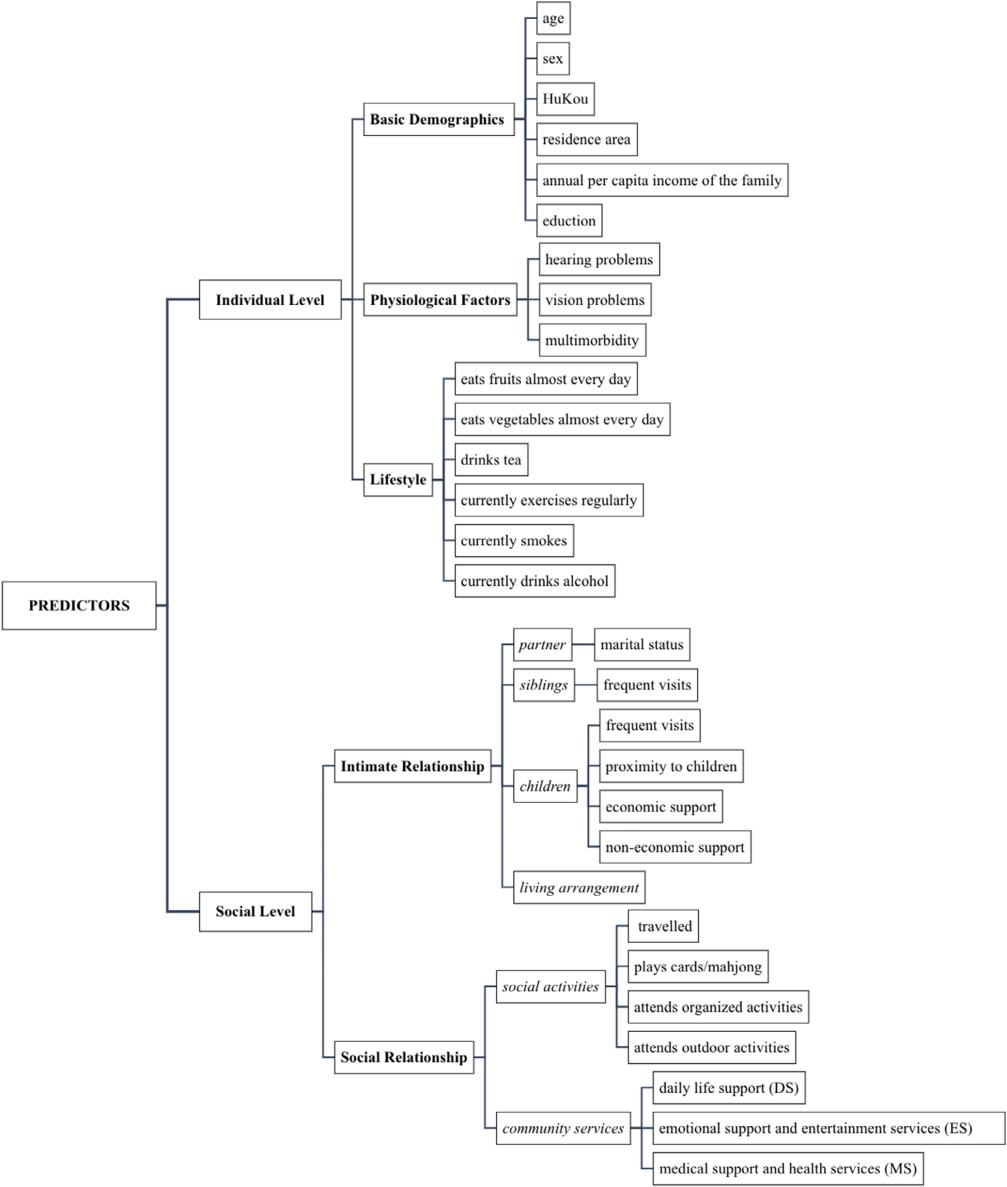
Theoretical framework: predictors of CDA patterns.

Figure 1 shows the theoretical framework. Individual‐level predictors, except for basic demographic information, include physiological factors (hearing, vision problems, and multimorbidity) and lifestyle variables (daily fruit and vegetable intake, smoking status, alcohol consumption, tea consumption, exercise), both of which have been found to be related to older adults' mental health (Fattouh et al., 2019; Frank et al., 2019; Lawrence et al., 2020; Zhang et al., 2021). For the social‐level factors, except for the direct measurements of intimate relationships, including partner, sibling, and child‐related factors (including geographical proximity, frequency, and forms of contact and support) (Park et al., 2005), the factor of living arrangement is also taken into account to indirectly reflect the family relationship (G.Y. Liu et al., 2012). Meanwhile, social relationships are reflected by participation in social activities and community services.

## METHODS

### Data and participants

The China Longitudinal Survey on Health and Longevity (CLHLS) is a nationwide and population‐based continuous survey jointly conducted by the Center for Healthy Ageing and Development at Peking University and the China Center for Disease Control and Prevention. Information including demographic, socioeconomic, and health‐related characteristics of people (aged ≥65) was collected across mainland China. A multistage disproportionate and random sampling method was used to recruit participants from approximately half of the counties and city districts in 23 Chinese provinces, which fully guarantees the representativeness of the sample (Center for Healthy Aging and Development Studies, 2020). The CLHLS has completed eight waves of investigations (1998, 2000, 2002, 2005, 2008–2009, 2011–2012, 2014, and 2017–2018) from 1998 to 2018, and approximately 113,000 individuals were involved. For living participants, information on demographic, socioeconomic, lifestyle, family structure, and physical and mental health characteristics was collected through self‐reported interviews. For deceased participants, information (e.g., chronic diseases, frequency of hospitalization, and activities of daily living) before death and the cause of death were collected by interviewing the elder's closest family member.

**FIGURE 2:**
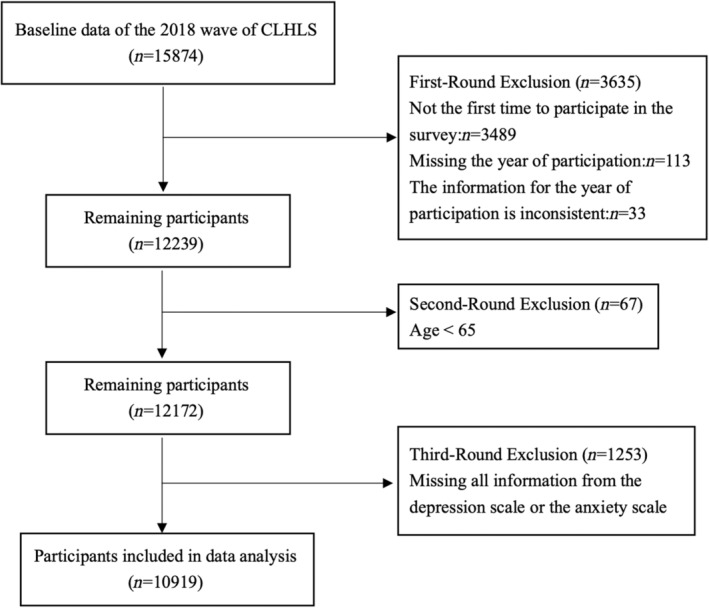
Flowchart for participant inclusion.

The present study selected cases from the dataset of 2018, because depression and anxiety of elderly people were only measured in the participant questionnaire in the 2018 survey. The inclusion criteria were as follows: (1) the participant only joined the CLHLS study in 2018 for the first time, because the survivor bias of participants from earlier waves may distort the results; (2) the participant was aged 65 years or above in 2018; (3) the participant responded to at least one item of CESD‐10 (the 10‐item Center for Epidemiologic Studies Depression Scale) and at least one item of GAD‐7 (the Generalized Anxiety Disorder 7‐item Scale) in the 2018 survey. After three rounds of screening by the first author, a total of 10,919 cases were included in the present study. The screening process is shown in Figure 2.

### Variables

Variables regarding participants' depression and anxiety and potential predictors listed in the theoretical framework (individual level and social level, see Figure 1) were extracted from the database.

#### Depression and anxiety

The CESD‐10, a self‐report measure to assess depressive symptoms within a week before the test (Andresen et al., 1994), was used to measure depression in the participants. The participants rated how often they experienced each symptom by choosing an item on a 4‐point Likert scale ranging from 0 (*never*) to 3 (*always*). After item 8 (Are you full of hope for future life?) and 9 (Do you feel as happy as you did when you were young?) are reverse‐scored, a total depression score can be calculated as the sum of all item scores. Higher scores show more severe depressive symptoms, and a total score of 10 or above indicates possible depression (Andresen et al., 1994). The CESD‐10 has been validated in older Chinese people (Yao et al., 2021).

The Generalized Anxiety Disorder 7‐item Scale (GAD‐7) was employed to measure participants' anxiety symptoms in the past 2 weeks (Yang et al., 2019). Participants reported how often they had experienced symptoms represented by items with a number ranging from 0 (*not at all*) to 3 (*almost every day*). A higher total score suggests greater anxiety symptoms. The cut‐off point of 5 distinguishes between no anxiety and mild anxiety (Spitzer et al., 2006). The GAD‐7 scale shows good reliability among the Chinese population (Shi et al., 2020).

#### Individual‐level predictors

Basic demographic variables included age (in years), sex (female vs. male), HuKou (urban vs. rural citizenship), residence area (city, town, or rural), annual per capita income of the family (<10,000 Yuan, 10,000–100,000 Yuan, >100,000 Yuan) and education (in years).

Physiological factors included hearing problems (yes vs. no), vision problems (yes vs. no), and multimorbidity. The factor of multimorbidity was coded as “yes” if the participant had two or more of the following 20 chronic conditions: hypertension, diabetes, heart disease, stroke, cerebrovascular disease, bronchitis, emphysema, asthma, pneumonia, pulmonary tuberculosis, cataracts, glaucoma, cancer, gastric or duodenal ulcer, Parkinson's disease, bedsore, arthritis, dementia, epilepsy, cholecystitis, cholelith disease, blood disease, rheumatism or rheumatoid disease, chronic nephritis, and hepatitis (Cheng et al., 2022). Four diseases (prostate tumour, uterine tumour, prostatic hyperplasia, mammary gland hyperplasia) that were also measured in the CLHLS were excluded for missing more than 20% of data.

Lifestyle was assessed by six health habits: having daily fruit and vegetable intake, drinking tea (yes vs. no), exercising frequently at present (yes vs. no), smoking at present (yes vs. no), and drinking alcohol at present (yes vs. no) (WHO Consultation, 2000). For fruit and vegetable intake, the answer of “*almost every day*” was coded as “yes,” and other answers (*often*, *occasionally*, or *rarely or never*) were coded as “no” (Li et al., 2020).

#### Social‐level predictors

Intimate relationships were measured in four aspects, as follows. (1) Partner: marital status (“yes” if the elder is currently married and living with a spouse or separated, and “no” for the answers of divorced, widowed, or never married). (2) Siblings: frequent visits by siblings (yes vs. no; this variable was retained owing to theoretical considerations, even though 27.8% of the data were missing). (3) Children: frequent visits by children (yes vs. no), proximity to children (“yes” if children live in the same village/neighborhood as or co‐reside with the elders), economic support, and noneconomic support. Specifically, economic support was reflected by the total money, including the cash and the value of goods offered to the elder in the last year by sons and their spouses, daughters and their spouses, and grandchildren and their spouses (in Yuan). According to the traditional Chinese value of filial piety, children's financial support reflects the care and filial respect for the elderly that is consistent with the expectation of “raising children to provide for old age” and the feedback model of family old‐age care, regardless of whether the elderly are actually economically poor or not (X.G. Liu, 2016). Existing literature shows that the closer the relationship between an elderly parent and their child in China, the greater the probability that the elderly person will receive financial support from their child (Ning & Wang, 2015; Shu et al., 2021). The noneconomic support has three indexes: the person whom the elder turns to first when having problems; the person whom the elder talks to most frequently in daily life; and the person whom the elder talks to first when they need to tell someone something. For the three indexes, answers of son, daughter, daughter‐in‐law, son‐in‐law, and grandchild and their spouses were coded as “yes” (Shu et al., 2021). (4) Living arrangement (with household members, alone, or in an institution).

Social relationships consist of social activities and community services. Social activity engagement was reflected by travel (times the participant toured beyond the home city or country in the past 2 years; the answer of “0” was coded as “no,” and the other times were coded as “yes”), playing cards/mahjong, attending organized activities, and attending outdoor activities. For the questions of playing cards/mahjong and attending organized activities, the answer of almost every day was coded as “yes,” while other options were coded as “no”; for the last question, if the answer was almost every day to any of the four items (including Tai Chi, square dancing, interact with friends, and other outdoor activities), this index was coded as “yes”.

Community‐based care services refer to a battery of social services provided to elderly people in the community, which include daily life support (DS; personal daily care life services or daily shopping), emotional support, entertainment services (ES; psychological consulting or social and recreation activities), and medical support and health services (MS; home visits or health education) (General Office of the State Council of the People's Republic of China, 2011; Yue et al., 2021). The category of service was coded as “yes” if any services within its category were available in the participants' community.

### Statistical analysis

First, descriptive analyses were run for depression and anxiety total scores and all predictors.

Then, LPA with unconditional models was conducted using all item scores on the CESD‐10 and GAD‐7 scales as observed responses to identify participants' CDA patterns. Models with 1 to 6 latent classes were examined among the 10,919 cases, and missing data were addressed with maximum likelihood estimation. To prevent convergence on solutions at a local maximum, we conducted the LPA with 1000 random starting values and 300 final‐stage optimizations. Model selection was based on statistical criteria and theoretical considerations. Statistical criteria included in the LPA were the Akaike information criteria (AIC; the smaller the better), sample‐size‐adjusted Bayesian information criteria (aBIC; the smaller the better), entropy (the higher the better – should be near 1), posterior probabilities (the higher the better – should be .80 or larger), the adjusted Lo–Mendell–Rubin likelihood ratio test results (LMR‐LRT; should be significant), the bootstrapped likelihood ratio test results (BLRT; should be significant) (Geiser, 2012), and the size of the classes (the size of the smallest class should be no less than 1% of the total sample).

Once the optimal number of groups was identified, we examined characteristics that defined each latent group by comparing the CESD‐10 and GAD‐7 item scores between the groups (analyses of variance, ANOVAs) and by comparing the total anxiety and depression scores within each group to the cut‐off points (*t* tests).

Then, bivariate analyses were conducted to compare class membership on individual‐level and social‐level predictors using Chi‐square tests for categorical predictors and ANOVAs for continuous predictors. All significant predictors in bivariate analyses were joined together in logistic regressions against latent class membership. All predictors except for “frequent visits by siblings” had less than 20% missing data, and the missing information of all variables is shown in Appendix Table S1. All missing data were addressed with multiple imputation. In the regressions, all categorical variables were turned into dummy variables, and the first level was used as the reference level.

Mplus 7.4 (Muthén & Muthén, 2014) was used to conduct LPA, and all remaining analyses were conducted in R 3.6.3 (R Core Team, 2020).

## RESULTS

### Sample characteristics

The average age of the 10,919 participants was 84.45 (range: 65–117, *SD* = 11.96), and 43.80% of them were males. Most of the participants had rural HuKou (69.80%) and lived with household members (79.80%). Nearly half of the samples' (48.20%) household total income was between 10,000 and 100,000 last year, and their average year of education was 2.98 (range: 0–22, *SD* = 4.21). On average, the total CESD‐10 and GAD‐7 scores were 9.46 (range: 0–28, *SD* = 4.16, Cronbach's alpha = .786) and 1.49 (range: 0–21, *SD* = 2.84, Cronbach's alpha = .919), respectively. Detailed information on all variables is shown in Table 1.

**TABLE 1:** Basic information of the 10,919 participants.

Category	Variables	*N*	*M* (*SD*)/%
Individual level–basic demographics	Age	10,919	84.45 (11.96)
	Sex	10,919	
	Male	4782	43.80
	Female	6137	56.20
	Category of HuKou	10,898	
	Urban	3273	30.00
	Rural	7625	69.80
	Category of residence	10,919	
	City	2758	25.30
	Town	3607	33.00
	Rural	4554	41.70
	Yearly income	9973	
	<10,000	2651	24.30
	10,000–100,000	5268	48.20
	>100,000	2054	18.80
	Education	9846	2.98 (4.21)
Individual level–physiological factors	Hearing problems	10,874	
	No	6801	62.30
	Yes	4073	37.30
	Vision problems	10,826	
	No	7177	65.70
	Yes	3649	33.40
	Suffer from multimorbidity	9597	
	No	5675	52.00
	Yes	3922	35.90
	Eat fruit almost every day	10,891	
	No	8381	76.80
	Yes	2510	23.00
	Eat vegetables almost every day	10,896	
	No	3738	34.20
	Yes	7158	65.60
	Drink tea	10,693	
	No	8800	80.60
	Yes	1893	17.30
	Smoke at present	10,801	
	No	9165	83.90
	Yes	1636	15.00
	Drink alcohol at present	10,747	
	No	9189	84.20
	Yes	1558	14.30
	Exercise regularly at present	10,769	
	No	7243	66.30
	Yes	3526	32.30
Social level–intimate relationship	Marital status	10,816	
	No	6105	55.90
	Yes	4711	43.10
	Proximity to children	10,393	
	No	3141	28.80
	Yes	7252	66.40
	Receive money from grandchildren	9044	
	No	6036	55.30
	Yes	3008	27.51
	Receive money from daughter or son‐in‐law	9283	
	No	3526	32.30
	Yes	5757	52.70
	Receive money from son or daughter‐in‐law	9534	
	No	3584	32.80
	Yes	5950	54.50
	Ask children first for help when having problems	10,666	
	No	3526	32.30
	Yes	7140	65.40
	Talk to children most frequently in daily life	10,641	
	No	5442	49.80
	Yes	5199	47.60
	Talk to children first when need to tell something	10,689	
	No	4591	42.00
	Yes	6098	55.80
	Frequent visits by children	10,481	
	No	512	4.70
	Yes	9969	91.30
	Frequent visits by siblings	7883	
	No	3369	30.90
	Yes	4514	41.30
	Living arrangement	10,834	
	With household member(s)	8709	79.80
	Alone	1725	15.80
	In an institution	400	3.70
Social level–social relationship	Traveled within the last 2 years	10,802	
	No	9344	85.60
	Yes	1458	13.40
	Play cards/mahjong	10,894	
	No	10,164	93.10
	Yes	730	6.70
	Attend organized activities	10,798	
	No	10,483	96.00
	Yes	315	2.90
	Attend outdoor activities	10,898	
	No	6888	63.10
	Yes	4010	36.70
	Daily life services (DS)	10,556	
	No	8872	81.30
	Yes	1684	15.40
	Emotional comfort and entertainment services (ES)	10,588	
	No	7658	70.10
	Yes	2930	26.80
	Medical support and health services (MS)	10,680	
	No	4760	43.60
	Yes	5920	54.20

### Latent CDA patterns

**FIGURE 3:**
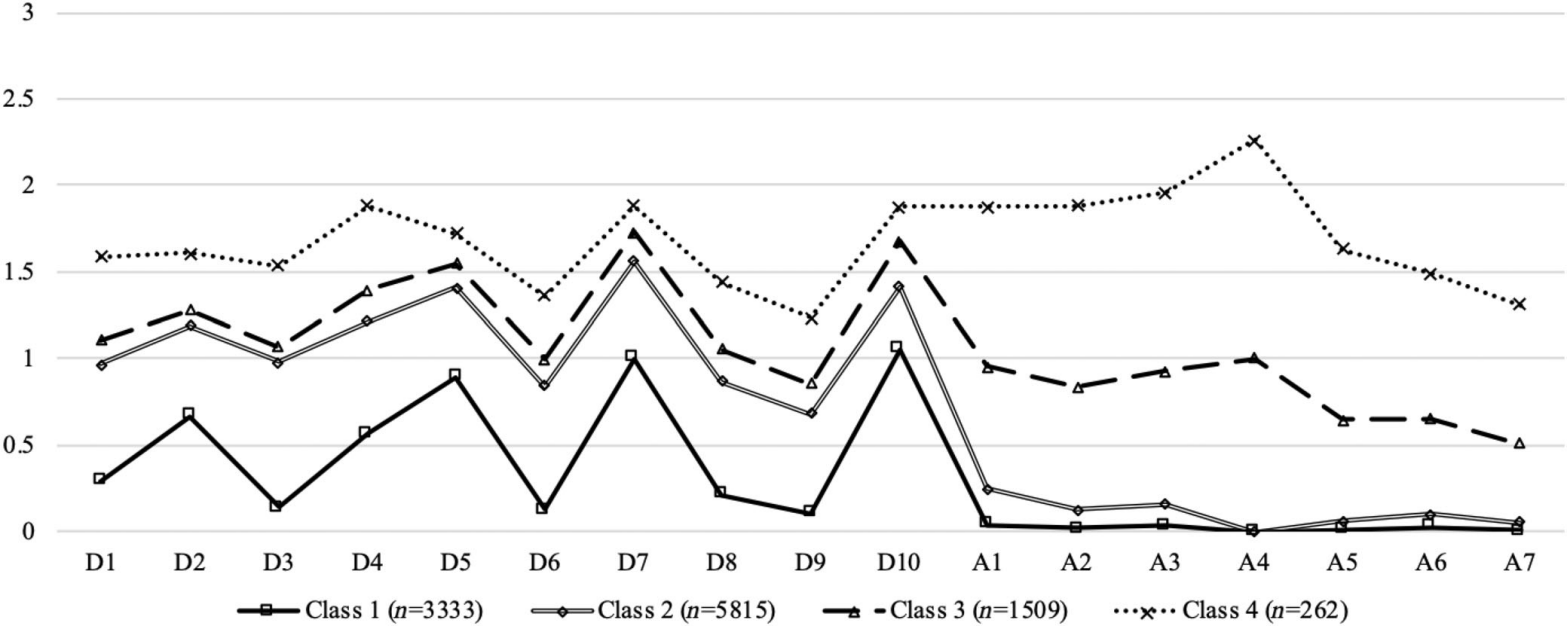
Symptoms profiles in the 4‐class model.Class 1: low symptoms of depression and anxiety; Class 2: mild depression only; Class 3: moderate depression and anxiety; Class 4: severe depression and anxiety. D1: worried about small things; D2: difficulty concentrating; D3: feeling depressed; D4: feeling of uselessness; D5: hopeful for future life; D6: nervous and scared; D7: feel happy like in young age; D8: feel lonely; D9: unable to continue life; D10: terrible sleep quality; A1: feeling annoyed; A2: uncontrolled worry; A3: worrying too much; A4: difficulty relaxing; A5: very anxious; A6: easily irritated; A7: feels like something terrible will happen.

The parameters of the 1‐class to 6‐class LPA models are shown in Table 2. The 4‐class model was selected (Figure 3): the entropy was satisfactory, and the AIC, aBIC, LMR‐LRT, and BLRT all suggested that it was better than the 2‐class and 3‐class models. Meanwhile, according to the LMR‐LRT results, the 4‐class model explained the data equally as well as the 5‐class model. Although the 6‐class model was better than the 5‐class model, it was given up for its relatively low entropy and high complexity.

**TABLE 2:** Parameters of LPA models with 1–6 classes.

No. of classes	AIC^a^	aBIC^a^	C_1_ ^a^	C_2_	C_3_	C_4_	C_5_	C_6_	Entropy	Posterior probabilities	LMR‐LRT^a^ ^,^ ^b^	BLRT^a^ ^,^ ^b^
1	328,055	328,195	10,919									
2	277,751	277,965	9444	1475					.981	.996, .984	***	***
3	261,693	261,981	8901	258	1760				.984	.996, .988, .978	***	***
4	237,690	238,052	3333	5815	1509	262			.983	.950, .960, .1, 1	***	***
5	230,241	230,678	1068	3209	5498	165	979		.936	1, .949, .952, .1, .961	.512	***
6	223,108	223,619	3189	717	713	5242	796	262	.946	.950, .975, .979, .956, .981, 1	***	***

^a^ AIC = Akaike information criteria; aBIC = sample‐size‐adjusted Bayesian information criteria; Cn = size of the nth group; LMR‐LRT = Lo–Mendell–Rubin adjusted LRT test; BLRT: bootstrapped likelihood ratio test.

^b^ *p < .05; **p < .01; ***p < .001.

In the selected model, 3333 (30.52%), 5815 (53.26%), 1509 (13.82%), and 262 (2.40%) cases were in Class 1, Class 2, Class 3, and Class 4, respectively. Between‐class differences in CESD‐10 and GAD‐7 item scores are shown in Table 3. From Class 1 to Class 4, depression and anxiety total scores increased gradually (*p* < .001). This trend was also true for most item scores. In terms of the CESD‐10 total score, only Class 1 was significantly lower than the cut‐off point (Class 1: *t* = −109.62, *p* < .001; Class 2: *t* = 31.00, *p* < .001; Class 3: *t* = 28.76, *p* < .001; Class 4: *t* = 18.56, *p* < .001). For GAD‐7 total scores, classes 1 and 2 were significantly lower than the cut‐off point (Class 1: *t* = −555.41, *p* < .001; Class 2: *t* = −234.83, *p* < .001), while classes 3 and 4 were significantly higher than the cut‐off point (Class 3: *t* = 8.80, *p* < .001; Class 4: *t* = 29.11, *p* < .001). Therefore, Class 1, Class 2, Class 3, and Class 4 were named “low symptoms of depression and anxiety,” “mild depression only,” “moderate depression and anxiety,” and “severe depression and anxiety,” respectively.

**TABLE 3:** Between‐class differences in CESD‐10 and GAD‐7 item scores and total scores.

	C1 (*n* = 3333)	C2 (*n* = 5815)	C3 (*n* = 1509)	C4 (*n* = 262)	*F*	Post hoc test
	*M* (*SD*)	*M* (*SD*)	*M* (*SD*)	*M* (*SD*)		
D1	0.29 (0.47)	0.97 (0.41)	1.11 (0.54)	1.59 (0.80)	2086.37***	1 < 2 < 3 < 4
D2	0.67 (0.77)	1.19 (0.56)	1.28 (0.64)	1.61 (0.79)	579.57***	1 < 2 < 3 < 4
D3	0.12 (0.33)	0.99 (0.34)	1.07 (0.51)	1.54 (0.75)	4416.87***	1 < 2 < 3 < 4
D4	0.56 (0.67)	1.22 (0.61)	1.40 (0.72)	1.88 (0.82)	1014.99***	1 < 2 < 3 < 4
D5	0.89 (1.02)	1.41 (0.76)	1.55 (0.74)	1.72 (0.79)	334.99***	1 < 2 < 3 < 4
D6	0.12 (0.34)	0.84 (0.48)	1.00 (0.54)	1.36 (0.79)	2222.50***	1 < 2 < 3 < 4
D7	0.99 (1.02)	1.56 (0.85)	1.73 (0.82)	1.88 (0.82)	355.17***	1 < 2 < 3 < 4
D8	0.21 (0.46)	0.87 (0.57)	1.05 (0.71)	1.44 (0.92)	1279.00***	1 < 2 < 3 < 4
D9	0.11 (0.36)	0.68 (0.57)	0.85 (0.60)	1.23 (0.82)	1180.89***	1 < 2 < 3 < 4
D10	1.05 (0.79)	1.41 (0.71)	1.67 (0.68)	1.88 (0.69)	346.89***	1 < 2 < 3 < 4
A1	0.04 (0.21)	0.25 (0.49)	0.96 (0.56)	1.87 (0.81)	2554.53***	1 < 2 < 3 < 4
A2	0.02 (0.15)	0.13 (0.37)	0.84 (0.56)	1.88 (0.86)	3442.85***	1 < 2 < 3 < 4
A3	0.03 (0.18)	0.16 (0.40)	0.93 (0.56)	1.96 (0.83)	3514.23***	1 < 2 < 3 < 4
A4	0.00 (0.00)	0.00 (0.00)	1.00 (0.00)	2.26 (0.44)	175919.54***	1 = 2 < 3 < 4
A5	0.01 (0.11)	0.06 (0.26)	0.65 (0.58)	1.63 (0.89)	3355.31***	1 < 2 < 3 < 4
A6	0.03 (0.17)	0.10 (0.32)	0.65 (0.58)	1.49 (0.91)	2298.16***	1 < 2 < 3 < 4
A7	0.01 (0.11)	0.05 (0.24)	0.51 (0.57)	1.31 (0.99)	2135.23***	1 < 2 < 3 < 4
CESD‐10	4.95 (2.53)	11.06 (2.4)	12.68 (3.41)	16.11 (4.71)	4525.82***	1 < 2 < 3 < 4
GAD‐7	0.14 (0.50)	0.75 (1.38)	5.53 (2.30)	12.40 (4.05)	9640.38***	1 < 2 < 3 < 4

*Note*: *p < .05; **p < .01; ***p < .001. D1: worried about small things; D2: difficult to concentrate; D3: feeling depressed; D4: feeling of uselessness; D5: hopeful for future life; D6: nervous and scared; D7: feel happy like young; D8: feel lonely; D9: unable to continue life; D10: terrible sleep quality; A1: feeling annoyed; A2: uncontrolled worry; A3: worried too much; A4: difficult to relax; A5: very anxious; A6: easily irritated; A7: feels like something terrible happens. CESD‐10: total scores of 10 items. GAD‐7: total scores of 7 items.

### Predictors of latent class membership

Table 4 displays the outcomes of bivariate analyses between all potential predictors and latent class membership. Chi‐square tests and ANOVAs detected no significant between‐class differences in frequent visits by siblings (*χ* ^2^ = 2.282, *df* = 3, *p* = .148), daily life services (DS; *χ* ^2^ = 2.593, *df* = 3, *p* = .052), or emotional comfort and entertainment services (ES; *χ* ^2^ = 1.165, *df* = 3, *p* = .322).

**TABLE 4:** Outcomes of bivariate analysis between latent class membership and every potential predictor.

Category	Variables	*n*	*χ* ^2^/*F*	*df*	*p*
Individual level–basic demographics	Age	10,919	14.882	3	.004**
	Sex	10,919	40.343	3	<.001***
	Category of HuKou	10,898	15.984	3	<.001***
	Category of residence	10,919	6.918	6	<.001***
	Yearly income per capita of the family	9973	18.926	6	<.001***
	Education	9846	37.752	3	.012*
Individual level–physiological factors	Hearing problems	10,874	16.365	3	<.001***
	Vision problems	10,826	64.231	3	<.001***
	Suffer from multimorbidity	9597	21.501	3	<.001***
	Eat fruit almost every day	10,891	62.242	3	<.001***
	Eat vegetables almost every day	10,896	48.798	3	<.001***
	Drink tea	10,693	22.554	3	<.001***
	Smoke at present	10,801	11.507	3	<.001***
	Drink alcohol at present	10,747	30.782	3	<.001***
	Exercise regularly at present	10,769	43.732	3	<.001***
Social level–intimate relationship	Marital status	10,816	16.775	3	<.001***
	Frequent visits by children	10,481	8.057	3	<.001***
	Proximity to children	10,393	2.787	3	.039*
	Receive money from grandchildren	9044	4.795	3	.004**
	Receive money from son or daughter‐in‐law	9534	6.033	3	<.001***
	Receive money from daughter or son‐in‐law	9283	7.397	3	<.001***
	Ask children first for help when having problems	10,666	9.768	3	<.001***
	Talk to children most frequently in daily life	10,641	10.296	3	<.001***
	Talk to children first when need to tell something	10,689	5.868	3	<.001***
	Frequent visits by siblings	7883	2.282	3	.148
	Living arrangement	10,834	4.179	6	<.001***
Social level–social relationship	Traveled within the last 2 years	10,802	19.657	3	<.001***
	Play cards/mahjong	10,894	17.475	3	<.001***
	Attend organized activities	10,798	8.812	3	<.001***
	Attend outdoor activities	10,898	40.047	3	<.001***
	Daily life services (DS)	10,556	2.593	3	.052
	Emotional comfort and entertainment services (ES)	10,588	1.165	3	.322
	Medical support and health services (MS)	10,680	5.385	3	.001**

* *p* < .05;

** *p* < .01;

*** *p* < .001.

The results of the multinomial logistic regression (*n* = 10,919) involving all significant predictors in bivariate analyses are shown in Table 5. Both individual‐level and social‐level factors have significant impacts on latent class membership.

**TABLE 5:** Findings of multinomial logistic regressions against latent class membership.

Category	Variables	Ref. low symptoms of depression and anxiety (*n* = 3333)			Ref. mild depression only (*n* = 5815)		Ref. moderate depression and anxiety (*n* = 1509)
		Mild depression only (*n* = 5815)	Moderate depression and anxiety (*n* = 1509)	Severe depression and anxiety (*n* = 262)	Moderate depression and anxiety (*n* = 1509)	Severe depression and anxiety (*n* = 262)	Severe depression and anxiety (*n* = 262)
		OR	OR	OR	OR	OR	OR
Individual level–basic demographics	Age	0.99**	0.97***	0.97***	0.98***	0.98*	1.00
	Male (ref. female)	1.05	1.48***	2.07***	1.40***	1.96***	1.40
	Education	0.99	0.98	0.98	0.99	0.99	1.00
	HuKou of rural (ref. urban)	1.17	0.88	1.09	0.75*	0.93	1.24
	Resident in the town (ref. city)	0.87	1.17	1.19	1.34*	1.37	1.02
	Resident in rural (ref. city)	0.78**	1.03	0.87	1.32*	1.12	0.84
	Yearly income in 10,000–100,000 (ref. <10,000)	0.86*	0.66***	0.59**	0.76***	0.69*	0.91
	Yearly income >100,000 (ref. <10,000)	0.93	0.65***	0.35***	0.70***	0.38***	0.54*
Individual level–physiological factors	Vision problems (ref. no)	1.50***	1.84***	1.90***	1.23**	1.28	1.03
	Hearing problems (ref. no)	1.10	1.31***	1.29	1.19*	1.17	0.98
	Suffer from multimorbidity (ref. no)	1.15**	1.71***	1.54**	1.49***	1.34*	0.90
	Eat fruit almost every day (ref. no)	0.74***	0.56***	0.46***	0.75**	0.62*	0.82
	Eat vegetables almost every day (ref. no)	0.78***	0.67***	0.76	0.86*	0.98	1.14
	Drink tea (ref. no)	0.85**	0.79*	1.27	0.93	1.48*	1.60*
	Exercise regularly at present (ref. no)	0.83***	0.90	0.73	1.08	0.88	0.82
	Smoke at present (ref. no)	0.96	1.06	0.54*	1.11	0.56*	0.50*
	Drink alcohol at present (ref. no)	0.71***	0.63***	0.91	0.88	1.27	1.44
Social level –intimate relationship	Married (ref. no)	0.85*	0.90	0.99	1.07	1.16	1.09
	Frequent visits by children (ref. no)	0.78*	0.59***	0.46**	0.76*	0.59*	0.78
	Proximity to children (ref. no)	1.03	1.02	1.03	0.98	0.99	1.01
	Receive money from son (ref. no)	1.10	1.01	1.25	0.91	1.13	1.23
	Receive money from daughter (ref. no)	1.00	1.25*	0.73	1.25**	0.73	0.58**
	Receive money from grandchildren (ref. no)	1.04	1.16	0.87	1.11	0.84	0.75
	Talk to the child most frequently (ref. no)	1.08	0.93	1.14	0.85	1.05	1.23
	Talk to the child first when need to tell something (ref. no)	0.84*	0.94	0.51**	1.12	0.61*	0.54*
	Ask the child first for help (ref. no)	0.97	0.94	2.38***	0.97	2.44***	2.51***
	Live alone (ref. with household member)	1.08	1.08	1.02	1.01	0.94	0.93
	Live in an institution (ref. with household member)	1.31*	1.53*	1.41	1.17	1.08	0.92
Social level–social relationship	Travel (ref. no)	0.90	0.89	0.78	0.99	0.87	0.88
	Play cards/mahjong almost every day (ref. no)	0.80*	0.71*	0.68	0.88	0.83	0.95
	Attend organized activities almost every day (ref. no)	0.77	1.40	0.87	1.80**	1.11	0.61
	Attend outdoor activities almost every day (ref. no)	0.86**	0.70***	0.46***	0.81**	0.53***	0.65*
	Medical support and health services (MS) (ref. no)	1.15**	1.00	0.89	0.87*	0.78	0.89

* *p* < .05;

** *p* < .01;

*** *p* < .001.

Compared with those reporting “low symptoms of depression and anxiety” (30.52%; *n* = 3333), those reporting “mild depression only” (53.26%; *n* = 5815) were more likely to have vision problems (odds ratio [OR] = 1.50, *p* < .001), suffer from multimorbidity (OR = 1.15, *p* = .006), live in an institution (OR = 1.31, *p* = .046), and have access to MS (OR = 1.15, *p* = .002), and were less likely to be older (OR = 0.99, *p* = .004), live in a rural area (OR = 0.78, *p* = .009), have a yearly income in the range 10,000–100,000 (OR = 0.86, *p* = .017), consume fruit (OR = 0.74, *p* < .001), vegetables (OR = 0.78, *p* < .001) or tea (OR = 0.85, *p* = .01), exercise (OR = 0.83, *p* < .001), drink alcohol (OR = 0.71, *p* < .001), be married (OR = 0.85, *p* = .038), have frequent visits by children (OR = 0.78, *p* = .031), talk to the child first when needing to tell something (OR = 0.84, *p* = .046), play cards/mahjong (OR = 0.80, *p* = .012), and attend outdoor activities (OR = 0.86, *p* = .004).

When comparing “moderate depression and anxiety” (13.82%; *n* = 1509) with “low symptoms of depression and anxiety,” most factors remained significant, except for living in rural areas, exercising, being married, talking to the child first when needing to tell something, and MS. In addition, those with “moderate depression and anxiety” were more likely to be male (OR = 1.48, *p* < .001), have hearing problems (OR = 1.31, *p* < .001), and receive money from daughters (OR = 1.25, *p* = .012). Furthermore, elderly individuals with “severe depression and anxiety” (2.40%; *n* = 262) were more likely to be male (OR = 2.07, *p* < .001), have vision problems (OR = 1.90, *p* < .001), suffer from multimorbidity (OR = 1.54, *p* = .004), and ask the child first for help (OR = 2.38, *p* < .001). In contrast, age (OR = 0.97, *p* < .001), yearly income in the range 10,000–100,000 (OR = 0.59, *p* = .003) or > 100,000 (OR = 0.35, *p* < .001), fruit intake (OR = 0.46, *p* < .001), smoke (OR = 0.54, *p* = .017), frequent visits by children (OR = 0.46, *p* = .004), talk to the child first when needing to tell something (OR = 0.51, *p* = .007), and attend outdoor activities (OR = 0.46, *p* < .001) reduced the risk of “severe depression and anxiety” compared with “low symptoms of depression and anxiety.”

With “mild depression only” being the reference group, those reporting “moderate depression and anxiety” were more likely to be male (OR = 1.40, *p* < .001), live in towns (OR = 1.34, *p* = .014) or rural areas (OR = 1.32, *p* = .029), have vision problems (OR = 1.23, *p* = .002), hearing problems (OR = 1.19, *p* = .012), suffer from multimorbidity (OR = 1.49, *p* < .001), receive money from daughters (OR = 1.25, *p* = .006), and attend organized activities (OR = 1.80, *p* = .001). Meanwhile, they were less likely to be older (OR = 0.98, *p* < .001), have rural HuKou (OR = 0.75, *p* = .012), have yearly income in the range 10,000–100,000 (OR = 0.76, *p* < .001) or > 100,000 (OR = 0.70, *p* < .001), eat fruit (OR = 0.75, *p* = .001) and vegetables (OR = 0.86, *p* = .02), have frequent visits by children (OR = 0.76, *p* = .039), attend outdoor activities (OR = 0.81, *p* = .003), and have access to MS (OR = 0.87, *p* = .032). Those who were male (OR = 1.96, *p* < .001), suffered from multimorbidity (OR = 1.34, *p* = .041), consumed tea (OR = 1.48, *p* = .033), and asked the child first for help (OR = 2.44, *p* < .001) were more likely to be in the “severe depression and anxiety” group than in the “mild depression only” group. In contrast, those who were older (OR = 0.98, *p* = .018), had a yearly income of 10,000–100,000 (OR = 0.69, *p* = .025) or > 100,000 (OR = 0.38, *p* < .001), ate fruit (OR = 0.62, *p* = .024), smoked (OR = 0.56, *p* = .025), were frequently visited by children (OR = 0.59, *p* = .037), talked to the child first when they needed to tell something (OR = 0.61, *p* = .042), and attended outdoor activities (OR = 0.53, *p* < .001) were more likely to be in the “mild depression only” group than in the “severe depression and anxiety” group. With “moderate depression and anxiety” as the reference group, elderly people in the “severe depression and anxiety” group were more likely to drink tea (OR = 1.60, *p* = .017), ask the child first for help (OR = 2.51, *p* < .001), and less likely to have a yearly income >100,000 (OR = 0.54, *p* = .028), smoke (OR = 0.50, *p* = .011), receive money from daughters (OR = 0.58, *p* = .005), talk to the child first when they needed to tell somebody something (OR = 0.54, *p* = .02), and attend outdoor activities (OR = 0.65, *p* = .016).

## DISCUSSION

In the present study, we investigated the CDA patterns of older adults in China and their predictors. A 4‐class solution best described our data. Specifically, those in the group “low symptoms of depression and anxiety” (30.52%; *n* = 3333) manifested the least depression and anxiety and did not meet the clinical diagnostic criteria, yet they had symptoms of anhedonia and restless sleep; those in the group “mild depression only” (53.26%; *n* = 5815) displayed mild depressive symptoms without anxiety; those in the group “moderate depression and anxiety” (13.82%; *n* = 1509) showed moderate symptoms of depression and anxiety; and those in the group “severe depression and anxiety” (2.40%; *n* = 262) demonstrated a high comorbid pattern of depression and anxiety. Both individual‐level and social‐level variables significantly predicted latent class membership.

To the best of our knowledge, this is the first LPA study to examine the patterns of CDA and their predictors with a large, nationally representative sample of elderly people in China. It is also the first analysis of this kind to base explorations of predictors on a systematic framework that involves both individual‐level and social‐level variables. In particular, detailed and comprehensive measurements of intimate relationships with children were involved, thus reflecting the cultural context of China that places great emphasis on intergenerational links.

### CDA profiles

In line with studies from other parts of the world (Curran et al., 2022; Lei et al., 2022), the present study also identified one group with no or low symptoms and one with high symptoms of depression–anxiety comorbidity. However, while approximately 50% of a sample of English people (aged ≥50) had no symptoms of either depression or anxiety even during the COVID‐19 period (Curran et al., 2022), nearly 70% of elderly people in our study had mental health problems. This emphasizes the urgent need for intervention among older Chinese adults. Meanwhile, instead of the “mild anxiety only” class identified in Ireland (aged ≥50) and the “mildly anxious” class found in Japanese general adults (*M*_age_ = 42.28 years) (Curran et al., 2020; Lei et al., 2022), the “mild depression only” class was unveiled in our study. These differences may partly be explained by the measurement tools. The 20‐item Center for Epidemiologic Studies Depression Scale (CESD‐20) was used in Curran's study and the Patient Health Questionnaire (PHQ‐9) was used in Lei's study, while the CESD‐10 was used in the present study. It was found that the prevalence of depression symptoms was higher when measured by the CESD‐10 instead of the 30‐item Geriatric Depression Scale (GDS‐30), Self‐Rating Depression Scale (SDS), CESD‐20, or PHQ‐9 (Tang et al., 2021). Moreover, the effect of age and the regional economic context should not be ignored. Previous studies have found that older people show more depression symptoms and fewer anxiety symptoms than younger people (Kim, 2010; Mirowsky & Schieman, 2008), and late‐life depression is more prevalent in developing countries (e.g., Nepal) than in developed countries (e.g., Europe and North America) (Thapa et al., 2018; Volkert et al., 2013).

When looking closely at each item, the symptoms of poor concentration, restless sleep, feelings of uselessness, and lack of positive affect were relatively salient in all profiles. This reflects the characteristics of elderly individuals in general. In addition to decreasing physical function accompanying the ageing process, such as sleep and concentration problems, the deterioration of social participation and contribution could bring feelings of uselessness and limit opportunities to experience positive emotions (Chen et al., 2022; Gruenewald et al., 2007; Ni et al., 2017; Y. Zhao et al., 2017). These symptoms commonly occur among elderly individuals and are associated with lower physical and psychological health and life satisfaction, as well as with higher mortality (Gu et al., 2016, 2017). Moreover, given that sleep problems were present in the “most healthy” group in the Japanese sample (Lei et al., 2022) but not in the “low or no symptoms” group in the English sample (Curran et al., 2022), the tendency of individuals from Eastern cultures to focus on somatic symptoms was demonstrated (Ryder & Chentsova‐Dutton, 2012). Meanwhile, it is worth noting that “difficult to relax” was the core symptom of anxiety, which is consistent with findings from exploratory graph analysis (Moriana et al., 2022). Therefore, such a symptom deserves serious attention in the prevention of and intervention for CDA in elderly Chinese people.

### Predictors of CDA profiles

Predictors of latent class membership also bring great insights. Consistent with previous variable‐oriented analyses, being male, and having younger age, low income, vision problems, hearing problems, and multimorbidity increased the risk of depression and anxiety (Curran et al., 2019; Curran et al., 2020; Frank et al., 2019; Gould et al., 2016; Huato & Chavez, 2021; Lawrence et al., 2020). Meanwhile, fruit and vegetable intake and exercise had the opposite effect, which echoed findings among elderly people in Hong Kong (aged ≥65) and Brazil (aged 60–69) (Chan et al., 2014; Figueira et al., 2023). However, unlike previous studies that found that lower education levels increase the likelihood of depression and anxiety (Curran et al., 2019), we found that the factor of education is insignificant: this may be due to the floor effect, in that our participants had low levels of education (*M*_education_ = 2.89 years).

In the present study, the effects of HuKou and place of residence are complicated. The contrasting findings regarding whether having an urban HuKou and living in the city may be jointly caused by urban dwellers' better economic conditions and infrastructure (Guo et al., 2018; L.W. Li et al., 2016; Sun & Lyu, 2020), as well as by their higher relative deprivation (Knight et al., 2009; Li & Liu, 2005; Lyu & Sun, 2020; Song & Kim, 2020). In terms of lifestyle, the protective effect of drinking alcohol may be related to its serving as a coping strategy after terrible life events and as a means of maintaining friendships (W. Sun et al., 2009). Meanwhile, the effect of smoking in reducing the risk of the “severe depression and anxiety” pattern may be due to the inversion of causality: baseline depression or anxiety was found to be associated with some type of later smoking behavior (Fluharty et al., 2017; Morrell & Cohen, 2006). In addition, the inconclusive effects of tea consumption may be because tea consumption was measured as a dichotomous variable, while types, frequencies, and doses were not assessed in detail.

How social‐level predictors, especially intimate relationships, influence CDA profiles is one of the novel explorations of the present study. People with married status in our study were more likely to have “low symptoms of depression and anxiety” rather than “mild depression only,” because a spouse can provide emotional gratification and relieve stress (Hajek & König, 2020). In contrast, the siblings' impacts were insignificant in our study. Because sibling contact and support decrease with age (P.‐C. Lu, 2007), the influence of the sibling relationship on one's mental health may not be as strong in old age. Meanwhile, findings regarding child‐related factors provide much insight. Older adults who were “frequently visited by children” and “talk to the children first when they need to share something” were more likely to have the “low symptoms of depression and anxiety” profile, showing how important intergenerational relationships are for the mental well‐being of older people (Silverstein et al., 2006). However, regarding “receiving money from daughter and son‐in‐law,” this factor increased the likelihood of “moderate depression and anxiety” compared with both “severe depression and anxiety” and “low symptoms of depression and anxiety” or “mild depression only.” On the one hand, elderly individuals who are supported by children usually feel proud and grateful (Abruquah et al., 2019; Hsu & Jones, 2012). On the other hand, according to the patrilocal tradition in China, the obligations of being parents' economic providers belong to sons rather than daughters and sons‐in‐law (Lin et al., 2003), so the money from daughters may make parents feel uncomfortable. In addition, older people who “ask the children first for help” were more likely to have the “severe depression and anxiety” profile. This may be explained by the inverted causal relationship: because older people in general do not seek assistance from children owing to a desire for independence and not to overburden children (Du et al., 2022), those who do so may indeed be in a worse state of life. In addition to direct measurements of relationships, the effects of living arrangements also reflect the importance of families. Living in an institution compared with living with household members is detrimental to mental health, while living alone has no such effect. Institutionalized elders may be viewed as being abandoned by their children, so they face not only the challenges of adjustment after leaving their community but also social stigma and cultural stereotypes (G. Y. Liu et al., 2012).

In terms of social relationships, playing cards or mahjong and attending outdoor activities significantly reduced the risk of “mild depression only” and “moderate depression and anxiety,” because leisure activities exerted a positive effect on the mental health of Chinese people aged ≥65 (Ross & Zhang, 2008; Teh & Tey, 2019). Meanwhile, it is worth noting that attending organized activities increased the risk of developing the “moderate depression and anxiety” profile rather than the “low symptoms of depression and anxiety” profile. This is probably because organized activities, especially compulsory activities, can bring additional obligations to older adults that cause mental distress (Tomioka et al., 2017). In addition, MS reduced the risk of “moderate depression and anxiety” compared with “mild depression only,” while it enhanced the likelihood of “mild depression only” compared with “low symptoms of depression and anxiety”. In general, medical support and health services can buffer older individuals from mental problems (Yue et al., 2021). However, because children are regarded as the primary source of care services for aged adults in China (You et al., 2020), communities may only intervene when there is a problem. Meanwhile, the insignificant effects of DS and ES showed that support from the community does not play a vital role in the mental health of older Chinese adults in general.

### Contribution

Being the first study to explore CDA patterns among a nationally representative sample in China, this research identified four groups with various patterns of CDA and unveiled salient symptoms across all groups. By clearly depicting CDA profiles, the findings promote theoretical understanding and lay the foundation for practical application in China. Meanwhile, through the investigation of predictors based on a systematic theoretical framework, how individual‐level and social‐level factors jointly shaped the CDA patterns in China was revealed: in addition to individual‐level impactors that are consistent around the world, social‐level factors, especially intimate relationships with partners and children rather than general links to siblings or the community, bring unneglectable impacts on whether and to what extent Chinese older adults may suffer from CDA in the cultural context of relationism, patriarchy, and filial piety. In addition to the implications that these identified influencing factors could bring to prevention and intervention plans, the analytical framework could inspire future studies on CDA among elderly individuals across the globe.

### Limitations

This study has several limitations. First, a cross‐sectional design was adopted, so causal inferences may not be strong for certain variables. Second, owing to the limits of the CLHLS dataset, certain individual‐level factors (e.g., religious‐related information) that have usually been examined in previous research were not addressed in the present study, and only individuals aged 65 years and above were included, although the age that defines an older person is usually 60 years in general (World Health Organization [WHO], 2021). Third, the outcome regarding the comparisons between the “severe depression and anxiety” and other classes should be treated with caution because the sample size of the “severe depression and anxiety” class is small.

### Practical implications

The findings of the present study can inform practice. First, because nearly 70% of older adults in China suffer from problematic CDA patterns, policymakers should respond to such a worrying situation and focus on intervention and prevention. Second, certain symptoms that are salient across all CDA patterns, such as restless sleep, lack of positive emotions, uselessness, and weak concentration, reflect difficulties and challenges faced by elderly Chinese adults in general and thus require serious attention, and “difficult to relax” needs to be focused on in dealing with profiles high in anxiety. Third, because findings show that social dimensions, especially intimate relationships with partners and children, are vital predictors of CDA patterns, preventing CDA among elderly individuals lacking such support is of great significance. After all, prevention is high on the agenda of mental health researchers, second only to treatment (Cuijpers et al., 2015). Moreover, it is crucial to involve the core family in formulating policies and developing social support systems to cope with ageing in China.

### Future direction

Future studies can explore the links and transformation mechanisms between the different CDA profiles of elderly Chinese individuals. Interactions between individual‐level and social‐level factors in the shaping of profile patterns are also worth examining. Moreover, cross‐cultural studies can be conducted to explore how culture‐specific variables influence CDA patterns and whether certain variables play different roles across different contexts.

## CONCLUSIONS

LPA identified four distinct CDA patterns among 10,919 older Chinese people: “low symptoms of depression and anxiety” (30.52%; *n* = 3333), “mild depression only” (53.26%; *n* = 5815), “moderate depression and anxiety” (13.82%; *n* = 1509), and “severe depression and anxiety” (2.40%; *n* = 262). While restless sleep, lack of positive emotions, uselessness, and weak concentration are salient across all profiles, “difficult to relax” is prominent in profiles high in anxiety. In addition to individual‐level variables, social‐level factors, especially intimate relationships with partners and children rather than general links to siblings or the community, have unneglectable impacts on whether and to what extent Chinese older adults suffer from CDA in the cultural context of relationism, patriarchy, and filial piety.

## CONFLICT OF INTEREST STATEMENT

The authors declare there are no conflicts of interest.

## ETHICS STATEMENT

No ethical approval was required for the present study because no new data were collected. The CLHLS study was approved by the Ethics Committees of Human Research at Peking University and Duke University (IRB00001052‐13074; Pro00062871). The application was sent to and granted by the Peking University Open Research Data Platform before data for the present study were acquired. All methods were carried out in accordance with relevant guidelines and regulations. All experimental protocols were approved by a named institutional and/or licensing committee. Informed consent was obtained from all subjects and/or their legal guardians, and details that might disclose the identity of the subjects under the study were omitted.

## Supporting information


**Table S1.** Missing information of the 10,919 participants.
