# Supplementary material for: Symptom patterns of comorbid depression and anxiety among older adults in China and their predictors
Source: Psych J. 2024 Jan 24;13(3):494–511. doi: 10.1002/pchj.729 (PMC11169763; doi:10.1002/pchj.729)
Supplement: Supplementary file 1 — Table S1. Missing information of the 10,919 participants. [file PCHJ-13-494-s001.docx]

**Appendices**

Table S1. Missing information of the 10919 participants.

| **Category** | **Variables** | **Missing cases** | **%** |
| --- | --- | --- | --- |
| CESD-10 | Worried about small things | 285 | 2.61 |
|  | Difficult to concentrate | 539 | 4.94 |
|  | Feeling depressed | 433 | 3.97 |
|  | Feeling of uselessness | 409 | 3.75 |
|  | Hopeful for future life | 772 | 7.07 |
|  | Nervous and scared | 415 | 3.80 |
|  | Feel happy like young | 769 | 7.04 |
|  | Feel lonely | 356 | 3.26 |
|  | Unable to continue life | 520 | 4.76 |
|  | Terrible sleep quality | 60 | 0.55 |
| GAD-7 | Feeling annoyed | 16 | 0.15 |
|  | Uncontrolled worry | 18 | 0.16 |
|  | Worried too much | 19 | 0.17 |
|  | Difficult to relax | 21 | 0.19 |
|  | Very anxious | 23 | 0.21 |
|  | Easily irritated | 23 | 0.21 |
|  | Feels like something terrible happens | 37 | 0.34 |
| Individual level-  basic demographics | Age | 0 | 0 |
|  | Sex | 0 | 0 |
|  | Category of Hukou | 21 | 0.19 |
|  | Category of residence | 0 | 0 |
|  | Yearly income | 946 | 8.66 |
|  | Education | 1073 | 9.83 |
| Individual level-  physiological factors | Hearing problems | 45 | 0.41 |
|  | Vision problems | 93 | 0.85 |
|  | Suffer from multimorbidity | 1322 | 12.11 |
|  | Eating fruits almost every day | 28 | 0.26 |
|  | Eating vegetables almost every day | 23 | 0.21 |
|  | Drinking tea | 226 | 2.07 |
|  | Smoke at present | 118 | 1.08 |
|  | Drink alcohol at present | 172 | 1.58 |
|  | Exercised regularly at present | 150 | 1.37 |
| Social level-  intimate relationship | Marital status | 103 | 0.94 |
|  | Proximity to children | 526 | 4.82 |
|  | Receive money from grandchildren | 1875 | 17.17 |
|  | Receive money from daughter or son in law | 1636 | 14.98 |
|  | Receive money from son or daughter in law | 1385 | 12.68 |
|  | Ask children first for help when having problems | 253 | 2.32 |
|  | Talk to children most frequently in daily life | 278 | 2.55 |
|  | Talk to children first when need to tell something | 230 | 2.11 |
|  | Frequent visits by children | 438 | 4.01 |
|  | Frequent visits by siblings | 3036 | 27.80 |
|  | Live arrangement | 85 | 0.78 |
| Social level-  social relationship | Traveled within the last two years | 117 | 1.07 |
|  | Played cards/mahjong | 25 | 0.23 |
|  | Attend organized activities | 121 | 1.11 |
|  | Attend outdoor activities | 21 | 0.19 |
|  | Daily life services (DS) | 363 | 3.32 |
|  | Emotional comfort and entertainment services (ES) | 331 | 3.03 |
|  | Medical support and health services (MS) | 239 | 2.19 |
